# FDG-PET is a good biomarker of both early response and acquired resistance in BRAF^V600^ mutant melanomas treated with vemurafenib and the MEK inhibitor GDC-0973

**DOI:** 10.1186/2191-219X-2-22

**Published:** 2012-05-31

**Authors:** Andreas R Baudy, Taner Dogan, Judith E Flores-Mercado, Klaus P Hoeflich, Fei Su, Nicholas van Bruggen, Simon-Peter Williams

**Affiliations:** 1 Department of Biomedical Imaging, Genentech Inc, 1 DNA Way, South San Francisco, CA, 94080, USA; 2 Department of Translational Oncology, Genentech Inc, 1 DNA Way, South San Francisco, CA, 94080, USA; 3 Department of Oncology, Roche Pharmaceuticals, 340 Kingsland, Street Nutley, NJ, 07110, USA

**Keywords:** Vemurafenib, GDC-0973, Melanoma, Drug resistance, Positron emission tomography

## Abstract

**Background:**

The BRAF inhibitor, vemurafenib, has recently been approved for the treatment of metastatic melanoma in patients harboring BRAF^V600^ mutations. Currently, dual BRAF and MEK inhibition are ongoing in clinical trials with the goal of overcoming the acquired resistance that has unfortunately developed in some vemurafenib patients. FDG-PET measures of metabolic activity are increasingly employed as a pharmacodynamic biomarker for guiding single-agent or combination therapies by gauging initial drug response and monitoring disease progression. However, since tumors are inherently heterogeneous, investigating the effects of BRAF and MEK inhibition on FDG uptake in a panel of different melanomas could help interpret imaging outcomes.

**Methods:**

^18^ F-FDG uptake was measured *in vitro* in cells with wild-type and mutant (V600) BRAF, and in melanoma cells with an acquired resistance to vemurafenib. We treated the cells with vemurafenib alone or in combination with MEK inhibitor GDC-0973. PET imaging was used in mice to measure FDG uptake in A375 melanoma xenografts and in A375 R1, a vemurafenib-resistant derivative. Histological and biochemical studies of glucose transporters, the MAPK and glycolytic pathways were also undertaken.

**Results:**

We demonstrate that vemurafenib is equally effective at reducing FDG uptake in cell lines harboring either heterozygous or homozygous BRAF^V600^ but ineffective in cells with acquired resistance or having WT BRAF status. However, combination with GDC-0973 results in a highly significant increase of efficacy and inhibition of FDG uptake across all twenty lines. Drug-induced changes in FDG uptake were associated with altered levels of membrane GLUT-1, and cell lines harboring RAS mutations displayed enhanced FDG uptake upon exposure to vemurafenib. Interestingly, we found that vemurafenib treatment in mice bearing drug-resistant A375 xenografts also induced increased FDG tumor uptake, accompanied by increases in Hif-1α, Sp1 and Ksr protein levels. Vemurafenib and GDC-0973 combination efficacy was associated with decreased levels of hexokinase II, c-RAF, Ksr and p-MEK protein.

**Conclusions:**

We have demonstrated that ^18^ F-FDG-PET imaging reflects vemurafenib and GDC-0973 action across a wide range of metastatic melanomas. A delayed post-treatment increase in tumor FDG uptake should be considered carefully as it may well be an indication of acquired drug resistance.

**Trial registration:**

ClinicalTrials.gov NCT01271803

## Background

Melanoma is the deadliest of all skin cancers, and its incidence has been steadily increasing over the past few decades, especially in Caucasians [1]. Advanced melanoma patients have an 8-month median time of survival, a figure that has improved little in the past decades despite enormous research efforts [2]. This, however, is beginning to change as personalized medicines targeting the most commonly mutated oncogenes are being evaluated in clinical trials. Vemurafenib is such an example and is an orally available ATP competitive inhibitor of the kinase domain within the BRAF oncogene [3]. BRAF is mutated in more than 50% of all melanomas, with BRAF^V600E^ being the predominant mutation, increasing the protein's kinase activity and, thereby, driving downstream cellular proliferation through the MAPK pathway [4]. BRAF^V600K/R^ mutations have also been reported to occur in melanoma but seem to be very rare and found mostly in other types of cancer [5].

Vemurafenib has shown impressive results in clinical trials of patients with BRAF^V600E^ mutations, resulting in almost complete tumor regression and increasing progression free survival by 7 months and has gained FDA approval [6]. However, two issues remain: tumors can become resistant to vemurafenib and regrow over time, and keratinocytes predisposed with mutated HRAS become highly proliferative due to paradoxical activation of the MAPK pathway, thereby, resulting in the development of squamous cell carcinomas in some patients [7,8]. It has recently been shown that the NRAS gene is highly susceptible to mutation upon continuous vemurafenib exposure in pre-clinical studies; mutations in MEK, itself, have also been reported [9-11]. Inhibition of BRAF combined with MEK should have the potential to address both outstanding issues, since MEK is a common downstream component of RAF and RAS signaling [12,13]. Evidence of positive results from dual BRAF and MEK inhibition in clinical trials is starting to emerge (vemurafenib and GDC-0973 currently in phase II), and effective evaluation of these drugs is important [14,15].

Positron emission tomography (PET) imaging using 2-deoxy-2-[fluorine-18]-D-glucose integrated with computed tomography (^18^ F-FDG-PET/CT) is a powerful tool in oncology imaging[16,17]. Sensitive detection is facilitated by the high metabolic demands of hyper-proliferating cells driving an increase of glucose uptake [18]. ^18^ F-FDG-PET/CT is primarily used in the clinic to stage and restage malignancies (determine tumor burden, evaluate drug efficacy) and to identify unknown metastases across a wide range of cancer types. In melanoma, it is applied in advanced and recurrent stages of the disease (American Joint Committee on Cancer stages III and IV) where it offers unparalleled levels of sensitivity and specificity relative to other techniques [19,20]. Despite the strengths of PET imaging, its clinical utility (i.e., its ability to inform patient management) depends strongly on the clinical setting due to differences among tumor types (composition, mutation status and glucose avidity) and a treatment's properties in altering tumor metabolism [21].

In order to guide the use of FDG-PET in the clinical development of novel anti-cancer therapeutics and further understand drug-tumor-imaging relationships, we ran a series of cell-based ^18^ F-FDG uptake assays *in vitro*. These employed a panel of melanoma cell lines and a robotic screening platform that allows for precise, reproducible, automated handling of the radioactive materials and subsequent optical and radioactivity readouts. We used these assays to assess the effects of MEK and RAF inhibition on FDG uptake across a wide range of melanomas, including the clinically relevant vemurafenib drug-resistant A375R lines with the expectation of recapitulating the responses seen in solid tumors with FDG-PET imaging.

## Methods

### Drug treatment and ^18^ F-FDG cell screening

All human cell lines were obtained from a standardized and curated in-house cell inventory (gCell; A375R1, R3 lines generated by Su et al. [10]) and grown in media either Dulbecco's modified eagle's medium (DMEM) or RPMI both supplemented with 10% of heat-inactivated FBS and 2 mM L-Glutamine (GIBCO, Life Technologies Corporation, NY, USA). Cells were plated on Cytostar-T (PerkinElmer Inc., MA, USA) scintillating microplates at a concentration of 25,000 cells/well on day 0. Vemurafenib and GDC-0973 (Hoffmann-La Roche, Ltd., NJ, USA) were dissolved in DMSO and added to media on day 1 and day 3. On day 4, 96 well plates containing cells were loaded into our automated Oasis robotics system for analysis: growth media was aspirated, cells washed once with Krebs-Ringer buffer (Sigma-Aldrich Corporation, St. Louis, MO, USA), then DMEM containing physiological glucose (100 mg/dL) and 2.7 μCi of 2-Deoxy-2 ^18^ F-FDG, and 5 μM Vybrant DyeCycle Ruby nuclear stain (Invitrogen #V-10273, Invitrogen Ltd., Renfrew, UK) was added per well and allowed to incubate for 1 hr. Plates were washed five times with Krebs-Ringer buffer, fixed in 4% paraformaldehyde, sealed, radioactivity measured using a Microbeta^2^ Plate counter (PerkinElmer Inc., MA, USA), then ruby fluorescence measured using a Synergy H4 Hybrid Multi-Mode Microplate reader (BioTek Instruments, Inc., VT, USA).

### Glucose transporter immunofluorescence

Cells were treated with drug as described above, then, were fixed in 4% paraformaldehyde; permeabilized with 0.2% Triton; blocked in 5% fish gelatin; incubated with either glucose transporter-1 (GLUT-1) antibody (Millipore #07-1401, Millipore Co., Billerica, MA, USA) or glucose transporter-3 (GLUT-3) antibody (Abcam #ab41525, Abcam Plc, Cambridge, UK) overnight; washed in PBS; incubated with secondary antibodies Alexa 488 (Invitrogen #A11008, Invitrogen Ltd.) or Cy3 (Jackson ImmunoResearch #715-165-150, Jackson ImmunoResearch Laboratories, Inc., PA, USA) and Hoechst nuclear dye; washed in PBS; and then analyzed by fluorescent microscopy.

### ^18^ F-FDG-PET/CT imaging

Athymic nude mice between 20-25 g in weight were supplied by Harlan Laboratories Inc. (WA, USA) and implanted in the right flank, subcutaneously with 5 million A375 or A375R1 vemurafenib resistant in 100 μl HBSS. When tumors reached a mean volume of 350-450 mm^3^, mice were imaged with a Siemens Inveon microCT/PET scanner (Siemens Medical Solutions, Inc., PA, USA) under light sevoflurane anesthesia (approximately 3.5%) for restraint only. Body temperature was maintained at 37°C by warm air flows under feedback control. The eyes were covered with ophthalmic ointment to prevent dehydration. Dynamic PET scans lasted 30 min, and X-ray CT scans were used for anatomical reference and attenuation correction. Blood glucose was measured pre- and post-scan; the average was used in subsequent calculations. List mode data were typically binned into 30 frames and reconstructed into images with 128 × 128 in-plane voxels of 0.4 × 0.4 mm and 0.8 mm through-plane voxel thickness using vendor-provided iterative OP-MAP implementation with the beta hyper-parameter set to 0.05. Animals were then randomized into three groups. Group 1 received 100 μl MCT vehicle, PO daily; group 2 received vemurafenib 50 mg/kg PO BID; and group 3 received vemurafenib 50 mg/kg BID PO as well as 7.5 mg/kg GDC-0973 PO daily for a total of 6 days and were imaged at baseline, day 3, and day 6 (*n* = 3-7 per group) [22]. Inveon Acquisition Workplace (Siemens Medical Solutions, Inc.) software was used to draw regions of interest which were defined as voxels within the tumor having at least 50% of the intensity of the brightest voxel within the tumor. This excludes the hypointense (possibly necrotic) core tissue. Time-activity data were exported to make Patlak-Gjedde plots [23] using liver as a blood reference tissue [24] using the statistical programming language R [25]. K_i_ is the tumor uptake rate constant for FDG, and MRGluc^MAX^ is the hypothetical maximum glucose uptake capacity [26] defined as K_i_ × ([blood glucose] + K_M_), where K_M_ is a half-saturation Michaelis constant set to 130 mg/dL; units = μmol/100 g/min [26]. All animal handling studies were conducted under the approval of Genentech's AALAC-accredited institutional animal care and use committee.

### Western blot and histological analysis

Tumors were excised on day 7 after the trial, one day after imaging, and frozen in liquid nitrogen fixed in formalin. Frozen tissue was lysed with RIPA buffer and then protein analyzed by western blot. Briefly, samples were reduced with β-mercaptoethanol in SDS buffer, heated to 95°C for 5 min, loaded into 8% Bis-Tris gels, blocked with milk/TBS-T and probed for hexokinase I and II (Santa Cruz #46695 and #6521, Santa Cruz Biotechnology, Inc., CA, USA), Hif-1α (BD transduction labs #610950, BD Biosciences, CA, USA), Sp1 (Cell Signaling #5931, Cell Signaling Technology, Inc., MA, USA), c-Raf and p-c-Raf (Millipore #04739 and #07-814, Millipore, Co.), Ksr (Santa Cruz #9317), Mek and p-Mek (Santa Cruz #6259 and Millipore #07-1474), Akt and p-Akt (Cell Signaling #9272 S and #9271 S), c-Myc (Sigma-Aldrich #C3956, Sigma-Aldrich Corporation) and β Actin (Sigma-Aldrich #A5441). For histological analysis, formalin-fixed tissue was transferred to 70% ethanol, paraffin embedded, sectioned at 6 microns and mounted on slides. Sections were then incubated with target retrieval (Dako #S1700, Dako Inc., CA, USA) at 99°C for 20 min, peroxidase activity was quenched with blocking solution (KPL #71-00-10, Kirkegaard & Perry Laboratories, Inc., MD, USA), endogenous avidin and biotin blocked (Vector Labs #SP-2001, Vector Laboratories, Inc., CA, USA) and endogenous immunoglobulins blocked with 10% normal goat serum in 3% BSA/PBS for 30 min. Slides were then incubated with anti-GLUT-1 (Lab Vision Thermo Scientific #RB-9052-P, Thermo Fisher Scientific Inc., MI, USA) for 1 hr, incubated with biotinylated goat anti-rabbit IgG H + L (Vector Labs #PK6100) for 30 min, incubated with ABC Elite Reagent (Vector Labs #PK6100), and finally developed with metal-enhanced DAB (Thermo Scientific #34065).

## Results

### *In vitro*^18^ F-FDG uptake demonstrates vemurafenib BRAF^V600E^ selectivity as well as striking MEK potentiation

**Figure 1:**
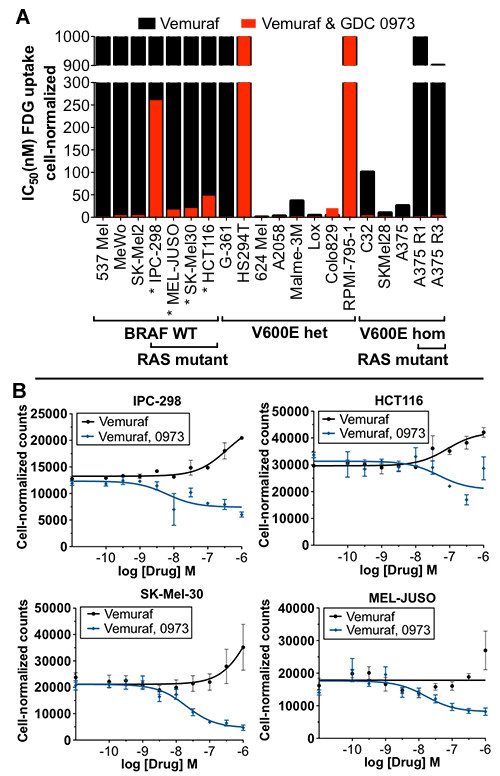
**BRAF and MEK inhibition of FDG uptake.** BRAF inhibition reduces FDG uptake associated with presence of BRAF^V600E^ mutations, while coadministration of MEK inhibitor broadly increases the effect. (**A**) A panel of melanoma cell lines that were homozygous null; heterozygous or homozygous positive for BRAF^V600^ mutations were treated with drug for 3 days, and FDG uptake was assessed (Asterisk, lines with increased FDG uptake from vemurafenib treatment). (**B**) Cell lines with both BRAF^V600E^ and RAS mutation were most susceptible to increased FDG uptake. Standard error of the mean shown, *n* = 4/group.

^18^ F-FDG uptake was evaluated in a panel of 19 melanoma cells, as well as the HCT 116 colorectal line, that had been treated with BRAF inhibitor vemurafenib alone, or in combination with MEK inhibitor GDC-0973 for a total of 3 days (Figure 1A). Vemurafenib was highly selective in reducing total FDG uptake within cell lines harboring one or two copies of mutant V600E BRAF allele but completely ineffective in BRAF WT lines as well as A375R1 and R3 vemurafenib-resistant cell lines. Coadministration with GDC-0973 resulted in significant decreases in ^18^ F-FDG uptake across almost all cell lines, independent of mutation status; the combination treatment strikingly overcame the A375R1 and R3 cell lines lack of response to vemurafenib alone. Similar IC_50_ values were found between total FDG uptake [Additional file 1 Figure S1A] and cell normalized FDG uptake (Figure 1A), indicating that the drug effects primarily induced changes in glucose transport and trapping rather than proliferation. The exception to this were the HS294T and RPMI-7951 melanomas, in which FDG response was induced by cell death rather than glucose metabolism; this was supported by the observation that vemurafenib and GDC-0973 were unable to induce changes in GLUT-1 at the membrane [Additional file 2: Figure S2].

### Vemurafenib induces increased ^18^ F-FDG uptake in BRAF WT-RAS mutants

Vemurafenib treatment was found to induce a 15% to 30% increase in FDG uptake in WT BRAF/RAS mutants (IPC-298 s, MEL-JUSO, SK-Mel-30 and HCT116) (Figure 1B). Comparison of total and cell-normalized values showed that the increase in FDG uptake was in part due to a proliferative effect induced by vemurafenib [Additional file 1: Figure S1B]. PET imaging experiments support this notion of continuous BRAF inhibition leading to an enhanced proliferative and metabolic response, as has previously been demonstrated *in vivo* treating HCT116 tumors with the BRAF inhibitor GDC-0879 Additional file 3: Figure S3] [27].

### Glucose transporter-1 membrane presence parallels vemurafenib and MEK-induced effects on FDG uptake

**Figure 2:**
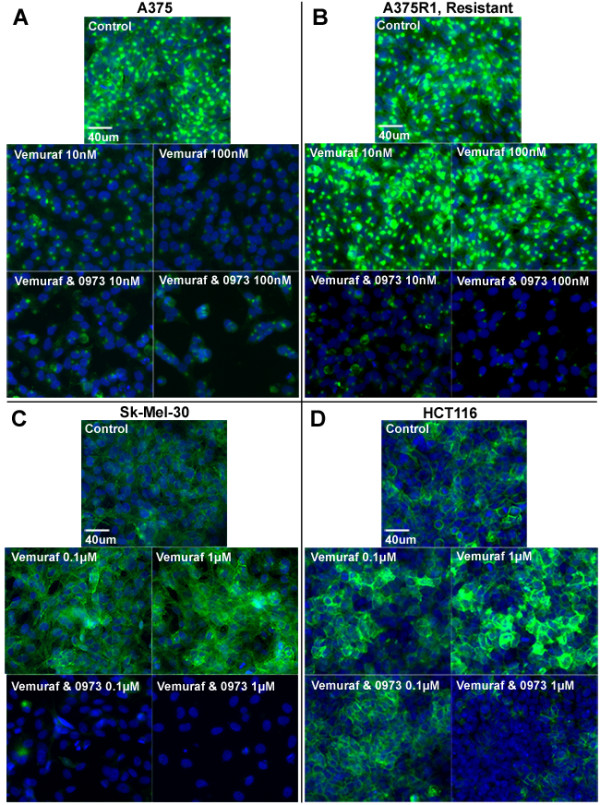
**BRAF and MEK modulation of GLUT-1.** BRAF and MEK inhibition results in changes in the amount of GLUT-1 at the cellular membrane associated with levels of FDG uptake. Immunofluorescent staining was performed for GLUT-1 (green) and nuclei (blue) on all panels of cells from Figure 1, which had been treated with drug for 3 days. (**A**) A375s, (**B**) resistant clone A375R1, (**C**) SK-Mel-30 melanomas and (**D**) HCT 116 colorectal cells.

Immunofluorescent staining for GLUT-1 and GLUT-3 showed that GLUT-1 was the major transporter present across the panel of 20 *in vitro* cell lines. GLUT-3, a secondary glucose transporter in melanomas, displayed no observable staining, suggesting that increased levels may only be detectable in some patient biopsies and cells transfected with high levels of the protein (GLUT-3 positive staining control; Additional file 4: Figure S4) [28]. Furthermore, GLUT-1 mRNA expression levels are significantly higher than GLUT-3 in most cancers, including melanoma, and appear to be the dominant protein in the process of FDG uptake (glucose transport) and trapping (hexokinase II) [Additional file 5: Figure S5]. The relative levels of GLUT-1 on the cellular membrane directly corresponded with the observed drug-induced changes on intracellular FDG uptake that was previously shown (Figure 2).

Vemurafenib treatment resulted in decreased levels of GLUT-1 on the cellular membrane across all BRAF^V600E^ lines in a dose-dependent manner (with the exception of HS294T and RPMI-7951; Additional file 3: Figure S3). Coadministration of the MEK inhibitor GDC-0973 significantly increased these effects and also overcame tumor vemurafenib resistance. The increased FDG uptake that is induced by vemurafenib treatment for the wild-type BRAF/RAS mutants could be attributed to the dose-dependent increases of GLUT-1 levels at the plasma membrane (Figure 2).

### FDG-PET is effective for monitoring BRAFi/MEKi efficacy and can be used to gauge vemurafenib-induced drug resistance

**Figure 3:**
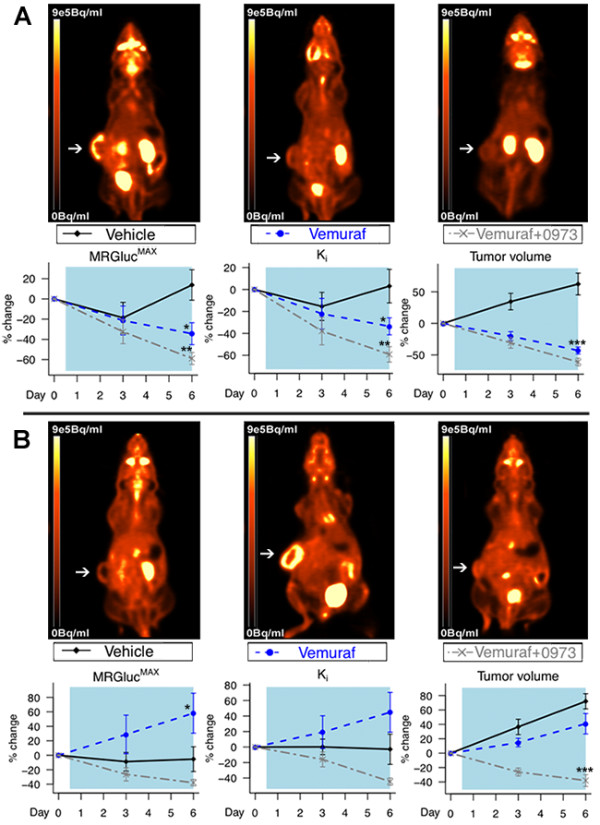
**FDG-PET imaging.** FDG-PET imaging is effective for monitoring vemurafenib and GDC-0973 combination drug action in BRAF^V600E^ mutant and resistant xenografts. (**A**) A375- and (**B**) A375 R1-resistant KRAS mutant melanomas were implanted in athymic nude mice and were administered vehicle, vemurafenib (50 mg/kg BID) or vemurafenib (50 mg/kg BID) and GDC-0973 (7.5 mg/kg QD). Dynamic FDG-PET imaging was performed at baseline, day 3 and day 6 after treatment. A reduction in K_i_ and MRGluc^MAX^ was induced on day 6 of imaging by both vemurafenib and 0973 combination treatment (Student's *t* test showing standard error of the mean A375: MRGluc^Max^ − vemuraf; *p = 0.02, combination; **p = 0.01, K_i_ − vemuraf; *p = 0.02, combination; **p = 0.001, tumor volume; ***p = 0.001. A375R1: MRGluc^Max^ − vemuraf; *p = 0.04, tumor volume; ***p = 0.001). White arrow points at the tumor.

**Figure 4:**
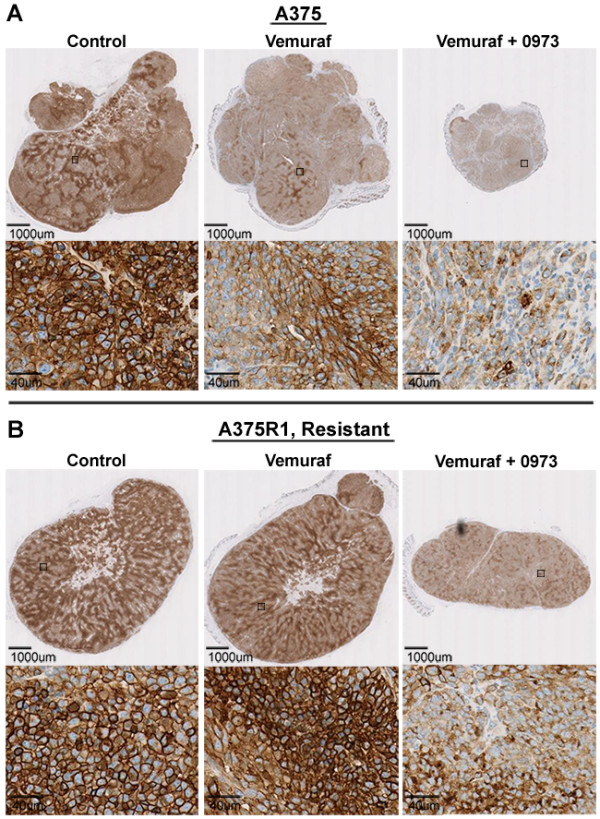
**Immunohistochemical staining of GLUT-1.** Vemurafenib treatment induces decreases in both total and membrane GLUT-1 staining in A375 tumors but not in the resistant A375R1 line. Immunohistochemical staining for GLUT-1 in (**A**) A375 tumor xenografts and (**B**) A375R1-resistant xenografts.

Vemurafenib treatment induced significant reductions in the dynamic FDG uptake parameters K_i_ and MRGLUC^MAX^ in A375 xenografts over the course of 6 days (Figure 3). Similar to earlier *in vitro* results, the addition of GDC-0973 also significantly improved FDG response. Both treatments also lead to reductions in tumor volumes and did not result in any significant changes in body weight. Vemurafenib treatment of the resistant A375R1 tumors resulted in a significant increase in dynamic FDG uptake over the period of drug treatment, indicating the effect as a sign of vemurafenib drug resistance. An increase in FDG uptake was also observed with 6 days of vemurafenib treatment *in vitro* [Additional file 6: Figure S6A] [10]. The addition of GDC-0973 overcame drug resistance as demonstrated by reductions in K_i_, MRGLUC^MAX^ and tumor volume. Histological analysis of the tumor xenografts demonstrated parallels between GLUT-1 membrane intensity and FDG uptake, and also confirmed the significant efficacy enhancement with the addition of GDC-0973 (Figure 4). Increases in GLUT-1 levels in vemurafenib-treated A375R1s were apparent using a more sensitive immunofluorescent histological approach [Additional file 6: Figure S6B].

**Figure 5:**
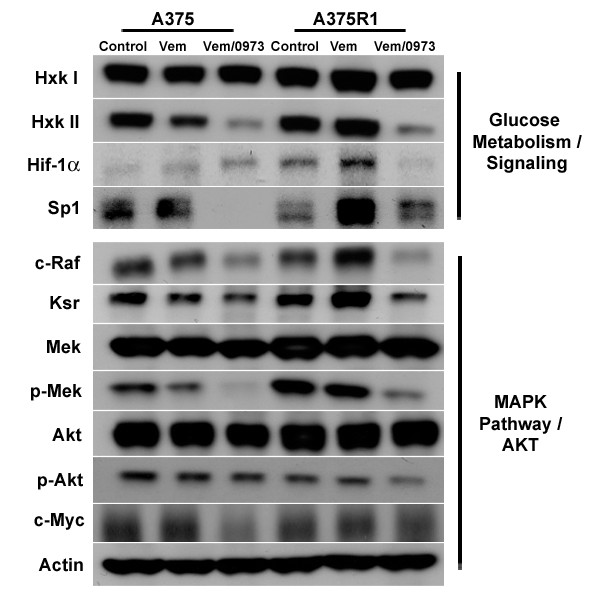
**Effects of BRAF and MEK on pathway proteins.** Vemurafenib treatment induced reductions of the MAPK pathway in A375s but caused increases in MAPK and glucose metabolism/signaling resistant A375 R1s. Western blot analysis of PET-imaged tumors that had been treated with drug for 6 days is shown.

Vemurafenib-resistant A375 R1 tumors exhibit increased baseline Hif-1α, whose levels are further increased by vemurafenib treatment along with Sp1 and Ksr, while FDG-PET efficacy is correlated with decreases in glucose metabolism and MAPK signaling. Following the last day of PET imaging, tumors were excised. Proteins involved with the FDG uptake as well as the MAPK and AKT pathways were measured by western blot (Figure 5). No major changes were found in hexokinase I between all groups; however, tumor predominant hexokinase II was decreased in both lines when treated with the drug combination. Hif-1α was faintly present in A375s but significantly expressed in A375R1s, and further induced by vemurafenib treatment in the resistant line but countered with combinatorial MEK inhibition. Sp1 levels were reduced by combination treatment in the A375 line, and reduced levels of vemurafenib induced Sp1 in the resistant line. c-RAF, p-MEK and Ksr protein levels were all reduced in both lines when treated with the RAF/MEK inhibitor drug combination. Drug combination also caused greater inhibitor effects on hexokinase II, CRAF and p-MEK expression in the A375 tumors than the A375 R1s with the exception in p-AKT that was only induced in the resistant line.

## Discussion

Vemurafenib is a personalized medicine that targets the product of a genetic mutation whose presence is required for therapeutic efficacy. The companion diagnostic used in patients in order to identify this mutation is the cobas® 4800 (F. Hoffmann-La Roche Ltd., USA) BRAF^V600^ test, which is a PCR-based procedure used on biopsy tissue isolated from a single melanoma lesion. A limitation with this approach is that advanced melanoma patients have dozens to hundreds of tumor lesions, which are likely to be genetically heterogeneous; therefore, a single biopsy does not assure that all lesions contain BRAF^V600^ mutations [29]. ^18^ F-FDG-PET imaging could perhaps discriminate between these populations relatively early in the course of treatment based on the effects of vemurafenib on the FDG-PET images. Importantly, our studies also suggest that an increase in FDG uptake observed in a specific tumor lesion from a patient on vemurafenib treatment could well be indicative of acquired drug resistance (Figure 3). If confirmed clinically, these findings could help inform decisions regarding discontinuation or changes in treatment, particularly since the increased FDG uptake is driven by metabolic changes that accelerate tumor growth rather than simply resulting in a lack of any response. We note that this phenomenon is a distinct metabolic adaption that differs from the well-known ‘flare’ phenomena observed by FDG-PET during the initial growth of tumors and ‘inflammatory flare’, which occurs during infection or T-cell activation [30].

We observed that the addition of a MEK inhibitor countered the vemurafenib-induced increases of glycolytic activity in WT-BRAF^V600^-Ras mutant populations. This is relevant since these populations may be present in sites not identified by the cobas® 4800 (F. Hoffmann-La Roche Ltd.) BRAF^V600^ test and would otherwise contribute to added cancerous growth (Figure 1) [13]. Additional advantages of combined RAF and MEK inhibition were the broader range of efficacy exhibited across melanoma cell lines and the ability to overcome vemurafenib drug resistance. While single GDC-0973 efficacy was not studied here, it is important to note that MEK inhibitors generally have higher toxicity profiles; therefore, maximizing the dose of targeted therapeutic vemurafenib is advantageous. This will be especially relevant in vemurafenib-resistant mutations that have acquired additional copy numbers of the BRAF gene [31,32].

The BRAF^V600E^ mutation has been reported to predict sensitivity to MEK inhibition, and RAS mutants have been found to be more resistant to treatment [33]. We find that the BRAF^V600E^ mutation was only slightly predictive of GDC-0973 response and only with regards to total FDG uptake rather than cell-normalized values; our findings are in agreement with the finding that RAS mutant cell lines have heightened resistance to MEK inhibition (Figure 1B; Additional file 1: Figure S1B). Other BRAF^V600^ cell lines have previously been shown to have high ^3^ H-FDG avidity relative WT lines, and vemurafenib has been shown to effectively reduce FDG uptake in a M248 BRAF^V600E^ xenograft mouse model [34]. We expand on these findings by demonstrating a clear BRAF^V600^ vemurafenib dose-response relationship by ^18^ F-FDG in a larger panel of distinct melanoma cell lines including the especially clinically relevant A375R1/R3 lines and validate effective *in vivo* FDG-PET response in A375 BRAF^V600E^ models.

GLUT-1 is the predominant glucose transporter facilitating enhanced glucose uptake in many tumor types and has been shown to be upregulated in isogenic cell lines when BRAF^V600E^ and KRAS^G13D^ mutations are introduced [35-38]. We did not observe any significant increases in baseline FDG uptake with the introduction of the RAS^K117N^ mutation alone in the A375R1 line relative to parental A375. We did, however, observe that in A375 R1 tumors, vermurafenib provoked the MAPK pathway via compensatory increases in c-RAF and Ksr; this likely lead to induction of Hif-1α and Sp1 transcription factors (independent of c-Myc), resulting in increased levels of hexokinase II, membrane GLUT-1 and, thereby, subsequent FDG uptake [39,40] (Figures 3B4B and 5; Additional file 6: Figure S6). Further evidence of compensatory increases in MAPK activation/growth by RAF inhibition was observed in treated BRAF-WT, RAS mutant HCT116 xenografts that displayed significantly increased tumor volumes and FDG uptake [Additional file 3: Figure S3].

## Conclusions

Acquired drug resistance may arise in patients taking vemurafenib for extended periods. Ongoing clinical trials combining vemurafenib with GDC-0973 seek to overcome this. Our study shows that ^18^ F-FDG can be a sensitive pharmacodynamic biomarker not only for assessing vemurafenib efficacy but also for acquired resistance. This could be a valuable early indicator of tumor rebound. Mechanistically, we find that this resistance is associated with the induction of membrane GLUT-1, likely driven by glycolytic regulators Hif-1α and Sp1. Furthermore, inhibition of MEK with GDC-0973 can prevent this metabolic tumor resistance signaling, and this too is recapitulated with FDG-PET imaging.

## Abbreviations

CT: Computed tomography; DMEM: Dulbecco's modified eagle's medium; GLUT-1: Glucose transporter-1; GLUT-3: Glucose transporter-3; PET: Positron emission tomography.

## Competing interests

The authors disclose that they are employees for Genentech, Inc., a member of the Roche group, and have no additional conflicts of interest.

## Authors’ contributions

AB executed the *in vitro* FDG uptake studies, immunostaining and drafted the manuscript. AB and JFM jointly carried out the *in vivo* FDG-PET imaging and analysis. AB and TD jointly performed protein extraction and western blot analysis. FS provided the A375 R1 and R3 vemurafenib-resistant cell lines, and provided manuscript input. KH, NvB and SW conceived of the study and helped draft the manuscript. All authors read and approved the final manuscript.

## Supplementary Material

Additional file 1 Total FDG uptake IC_50_ values from Figure 1A and B that have not been normalized to cell number. Click here for file

Additional file 2 Vemurafenib, as well as GDC-0973 combination treatments did not result in any apparent changes of GLUT-1 levels (green) in RPMI-795 1 and HS294T melanomas. Click here for file

Additional file 3 GDC-0879 BRAF inhibitor increase in FDG uptake and tumor volume *in vivo* in HCT 116 colorectal (BRAF WT, RAS mutant) tumor xenografts. Click here for file

Additional file 4 GLUT-3 immunofluorescent staining in H1299 cells. H1299 cells were transiently transfected with mock empty vector or CMV-Glut3 vector for 3 days, then fixed and stained for GLUT-3. Click here for file

Additional file 5 Genomic mRNA expression levels taken from an integrated set of gene data from Entrez, Ensembl and Genentech databases. Glucose transporters 1 and 3, and hexokinase II mRNA expression levels are shown in normal and cancer tissues across a range of tumor types. Click here for file

Additional file 6 Six days of vemurafenib exposure results in increased FDG uptake in A375R1 resistant cells *in vitro* and induces upregulation of GLUT-1 in A375R1 xenograft sections. (**A**) Continuous treatment of 1nM vemurafenib alone or in combination with 1nM GDC-0973 effects on FDG uptake. Student's *t* test showing standard error of the mean. **p < 0.01, ***p < 0.001 B. Vemurafenib induces increase in total membrane GLUT-1 expression (green) in immunofluorescently stained sections (blue = hoechst nuclear stain), shown at 4× magnification. Click here for file
