# Supplementary figures and images for: FDG-PET is a good biomarker of both early response and acquired resistance in BRAFV600 mutant melanomas treated with vemurafenib and the MEK inhibitor GDC-0973
Source: EJNMMI Res. 2012 May 31;2:22. doi: 10.1186/2191-219X-2-22 (PMC3405466; doi:10.1186/2191-219X-2-22)

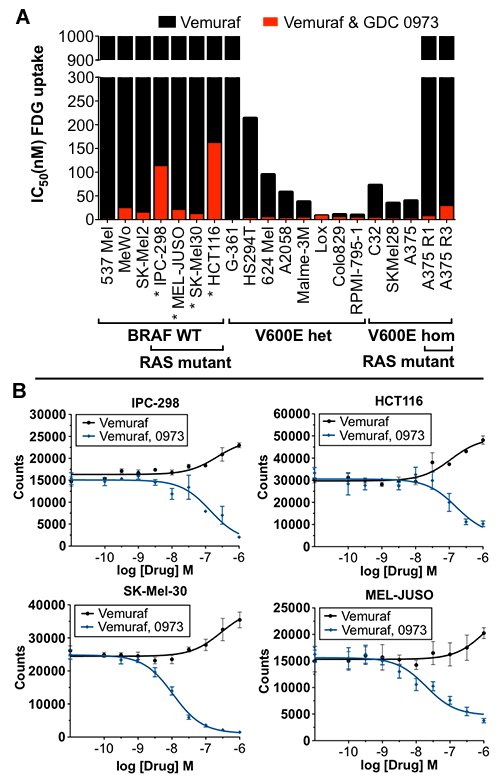

Supplement: Additional file 1 — Total FDG uptake IC50 values from Figure 1A and B that have not been normalized to cell number. [file 2191-219X-2-22-S1.tiff]

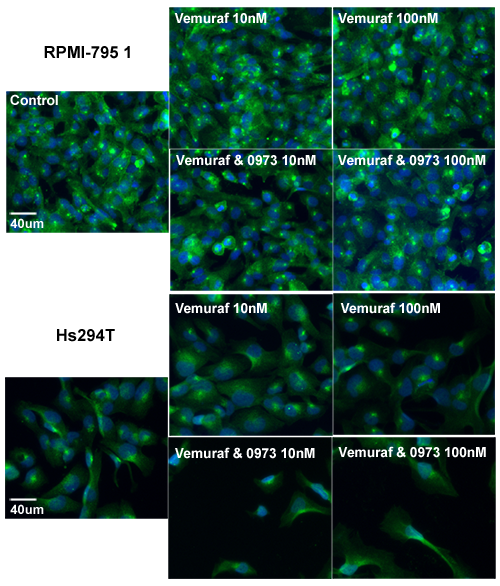

Supplement: Additional file 2 — Vemurafenib, as well as GDC-0973 combination treatments did not result in any apparent changes of GLUT-1 levels (green) in RPMI-795 1 and HS294T melanomas. [file 2191-219X-2-22-S2.tiff]

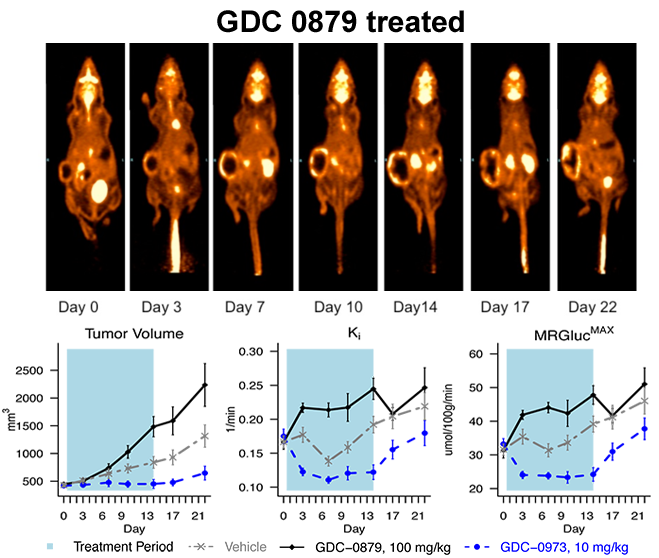

Supplement: Additional file 3 — GDC-0879 BRAF inhibitor increase in FDG uptake and tumor volume in vivo in HCT 116 colorectal (BRAF WT, RAS mutant) tumor xenografts. [file 2191-219X-2-22-S3.tiff]

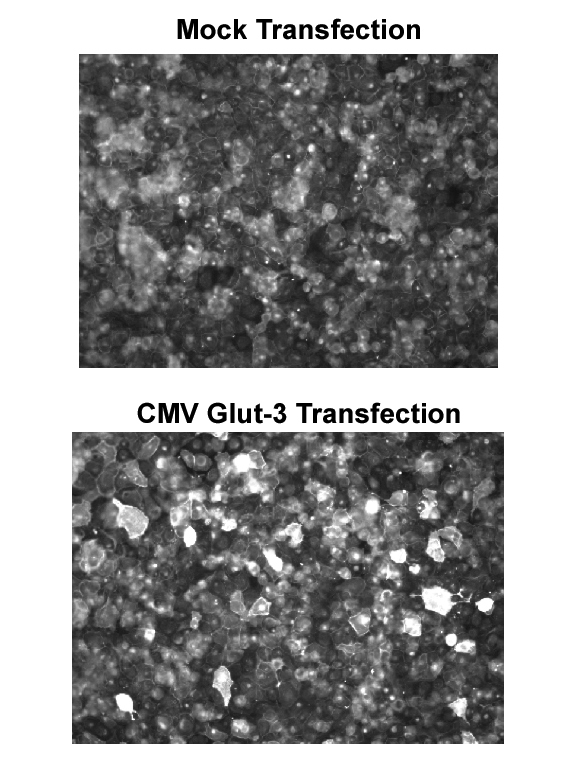

Supplement: Additional file 4 — GLUT-3 immunofluorescent staining in H1299 cells. H1299 cells were transiently transfected with mock empty vector or CMV-Glut3 vector for 3 days, then fixed and stained for GLUT-3. [file 2191-219X-2-22-S4.tiff]

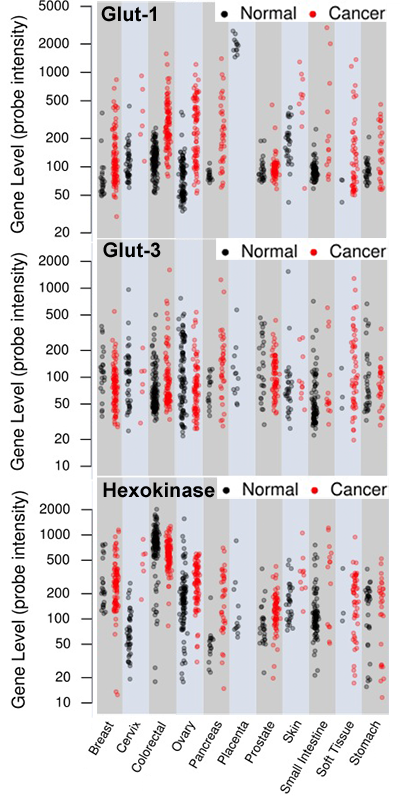

Supplement: Additional file 5 — Genomic mRNA expression levels taken from an integrated set of gene data from Entrez, Ensembl and Genentech databases. Glucose transporters 1 and 3, and hexokinase II mRNA expression levels are shown in normal and cancer tissues across a range of tumor types. [file 2191-219X-2-22-S5.tiff]

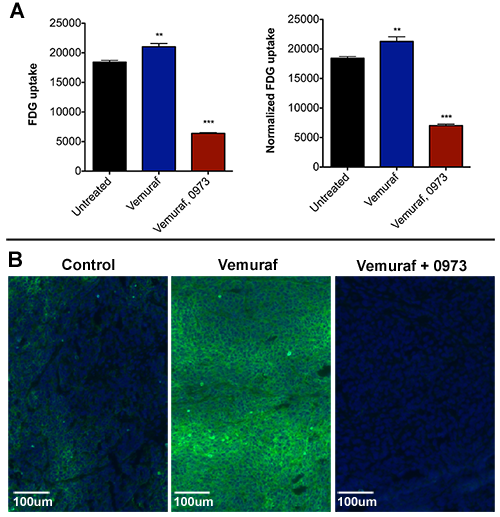

Supplement: Additional file 6 — Six days of vemurafenib exposure results in increased FDG uptake in A375R1 resistant cells in vitro and induces upregulation of GLUT-1 in A375R1 xenograft sections. (A) Continuous treatment of 1nM vemurafenib alone or in combination with 1nM GDC-0973 effects on FDG uptake. Student's t test showing standard error of the mean. **p < 0.01, ***p < 0.001 B. Vemurafenib induces increase in total membrane GLUT-1 expression (green) in immunofluorescently stained sections (blue = hoechst nuclear stain), shown at 4× magnification. [file 2191-219X-2-22-S6.tiff]
